# OMMR: Co-registration toolbox of OPM-MEG and MRI

**DOI:** 10.3389/fnins.2022.984036

**Published:** 2022-09-15

**Authors:** Fuzhi Cao, Nan An, Weinan Xu, Wenli Wang, Wen Li, Chunhui Wang, Yanfei Yang, Min Xiang, Yang Gao, Xiaolin Ning

**Affiliations:** ^1^Zhejiang Provincial Key Laboratory of Ultra-Weak Magnetic-Field Space and Applied Technology, Hangzhou Innovation Institute, Beihang University, Hangzhou, China; ^2^Key Laboratory of Ultra-Weak Magnetic Field Measurement Technology, Ministry of Education, School of Instrumentation and Optoelectronic Engineering, Beihang University, Beijing, China; ^3^Research Institute for Frontier Science, Beihang University, Beijing, China; ^4^Beijing Academy of Quantum Information Sciences, Beijing, China

**Keywords:** magnetoencephalography (MEG), OPM-MEG, co-registration, electromagnetic digitization system, optical scanner

## Abstract

Magnetoencephalography (MEG) based on optically pumped magnetometers (OPM-MEG) has shown better flexibility in sensor configuration compared with the conventional superconducting quantum interference devices-based MEG system while being better suited for all-age groups. However, this flexibility presents challenges for the co-registration of MEG and magnetic resonance imaging (MRI), hindering adoption. This study presents a toolbox called OMMR, developed in Matlab, that facilitates the co-registration step for researchers and clinicians. OMMR integrates the co-registration methods of using the electromagnetic digitization system and two types of optical scanners (the structural-light and laser scanner). As the first open-source co-registration toolbox specifically for OPM-MEG, the toolbox aims to standardize the co-registration process and set the ground for future applications of OPM-MEG.

## Introduction

Magnetoencephalography (MEG) is a non-invasive, functional imaging technique that measures magnetic fields generated by neural activity in the brain (Hämäläinen et al., 1993). The recent emergence of optically pumped magnetometers (OPM) (Kominis et al., 2003; Borna et al., 2017) based MEG (OPM-MEG) overcomes the limitations of conventional superconducting quantum interference devices (SQUID) based MEG system (SQUID-MEG), by operating without a cryogenic dewar as well as closer to the subject’s scalp. This results in lower cost and higher signal strength for OPM-MEG (Iivanainen et al., 2017; Boto et al., 2018). Furthermore, flexible sensor configuration makes OPM-MEG a promising tool, as it can be adjusted to fit all ages and various measurement scenarios (Hill et al., 2019; Roberts et al., 2019; Tierney et al., 2021).

Source localization for OPM-MEG investigates the neural origin of the brain and has a wide application in neuroscience (Boto et al., 2021; Seymour et al., 2021b) and clinical research (Liang et al., 2021; Feys et al., 2022). Reliable source localization results presuppose the interference suppression technique (Seymour et al., 2021a), accurate co-registration of the OPM-MEG and magnetic resonance imaging (MRI) (Zetter et al., 2018), and source imaging methods (An et al., 2022a). Previous research has resulted in useful open-source software such as FieldTrip (Oostenveld et al., 2011), MNE-Python (Gramfort et al., 2014), and SPM (Litvak et al., 2011), which contain codes and algorithms for data preprocessing and localization for OPM-MEG promoting collaboration and communication in the research community. However, an open-source toolbox specifically for the co-registration of OPM-MEG and MRI has not been provided so far, which limits the application of OPM-MEG.

Through co-registration, the accurate sensor positions and orientations relative to the cortical surface are obtained, and used to describe the geometrical relationship, between brain sources and measured fields, when solving the forward problem. The co-registration of OPM-MEG and MRI is different than that of SQUID-MEG as the sensors for OPM-MEG are visible and have more flexible configurations, thereby making the co-registration of OPM-MEG and MRI a complex and difficult task, especially for researchers and clinicians with no programming experience.

Previously, we focused on the implementation of co-registration methods for OPM-MEG based on three commonly used devices: the electromagnetic digitization system (Fastrak), structured-light scanners, and laser scanners and quantified their co-registration accuracy (Cao et al., 2021). Further, OMMR toolbox on Matlab was developed to conveniently accomplish the co-registration process of OPM-MEG. Each of the co-registration methods provided in the OMMR toolbox has its own advantages and disadvantages. The details of the characteristics and absolute accuracy of each co-registration method have been shown in our previous work (Cao et al., 2021). For a brief summary, the co-registration accuracy values obtained through the reference phantom experiment were as follows: (1) For the laser scanner, the location and orientation errors were 0.72 mm and 0.18^°^, respectively; (2) for Fastrak, 1.22 mm and 0.27^°^, respectively; (3) for the structured-light scanner, 2.19 mm and 0.91^°^, respectively. The purchase cost in descending order is (1) laser scanner; (2) Fastrak; (3) structured-light scanner. The time consumptions in the experiment in decreasing order were as follows: (1) Fastrak (3 min 44 s); (2) structured-light scanner (3 min); (3) laser scanner (1 min 30 s). The Fastrak is the most commonly used device in the SQUID-MEG and has additional functions, for example, recording the head movement of the subject. In this toolbox, we provide all the solutions for these devices, and users can choose one of the co-registration methods according to their practical needs.

The toolbox is expected to standardize and facilitate the co-registration of OPM-MEG and MRI, by integrating the co-registration methods for each device and by being adaptable to practical applications. In addition, OMMR provides an easy to use graphical user interface (GUI) to facilitate its use. By doing so, it is expected that OMMR will contribute to the increased adoption of OPM-MEG applications.

## Toolbox overview

### Co-registration device

The OMMR toolbox provides co-registration methods corresponding to three commonly used devices, including the electromagnetic digitization system (Fastrak) (Boto et al., 2022), structured-light scanners (Zetter et al., 2019; Hill et al., 2020), and laser scanners (Hironaga et al., 2014; Ebrahim M. A. B., 2015). These devices are used to digitize, or scan, the face or helmet, in such cases. Each of the devices has its own advantages and disadvantages and they can be flexibly selected according to the practical requirements. The Fastrak system contains a transmitter and a receiver (stylus). The transmitter generates alternating magnetic fields and the stylus detects this fields (Koessler et al., 2007). The position and orientation of the stylus relative to the transmitter are then computed. During co-registration, the stylus can be used to digitize the space points such as the face and scalp points. A structured-light scanner projects structured light onto the scanned object and uses one or more cameras to capture it. This generates three-dimensional (3D) colored images through the changes in the pattern captured. Finally, a laser scanner includes two cameras and utilizes one, or several, diode lasers to project a cross on the scanned surface. The distances between the cameras and the laser are known, such that the position of the projected crosshair can be triangulated. Based on this, the scanner calculates the distance from the object and obtains the 3D point cloud data.

### Supported functions

**FIGURE 1:**
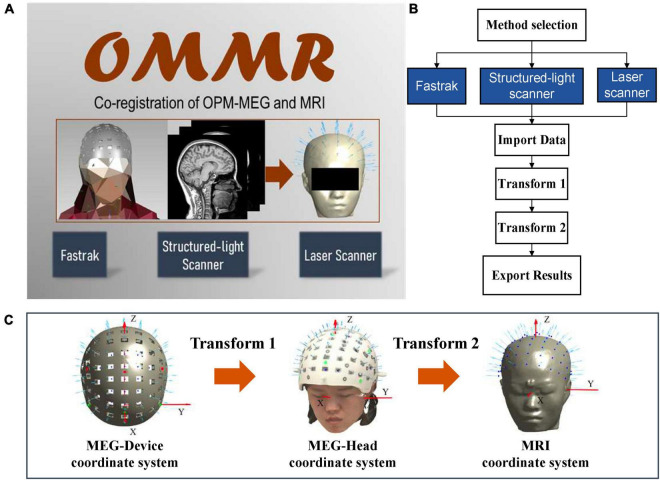
**(A)** Homepage of OMMR; **(B)** flow chart of the OMMR toolbox (see visualization 1); **(C)** involved coordinate systems.

The homepage of the OMMR toolbox provides a selection of the different devices, as shown in Figure 1A. When one of the devices is selected, the page will forward to the pipeline co-registration process for the corresponding device (Figure 1B). Although, there are some differences in the co-registration methods of the three devices, they all involve a two-step transformation of the sensor positions and orientations in the three coordinate systems. As shown in Figure 1C, transform 1 and transform 2 involve the transformations from MEG-Device to MEG-Head and MEG-Head to the MRI coordinate system, respectively.

### Data input and output

After selecting an appropriate device, the first step of the co-registration process is to import the required data. For the co-registration, the required data are collected in different coordinate systems. The needed data and their corresponding file formats are summarized in Table 1.

**TABLE 1:** Input data during co-registration.

Device	Fastrak	Structured-light scanner	Laser scanner
MEG-device	Sensors data (sensor.mat); helmet reference points (ref.mat)		Sensors data (sensor.mat); helmet data (helmet.ply)
MEG-head	Head points (head points.xlsx); Helmet reference points (helmet ref points.xlsx)	3D scan (scan.ply)	3D scan (laser_scan.ply)
MEG-MRI	MRI (MRI.ply)		

#### Input data in magnetoencephalography-device coordinate system

When the helmet is designed, the data obtained in the MEG-Device coordinate system, include (1) the initial setting positions and orientations of sensors (sensor.mat); (2) the positions of the reference points (ref.mat), for example, the green and red points on the designed helmet (Figure 1B); and (3) the polygon file of the 3D helmet (helmet.ply). The sensor.mat data is required for all devices and they will be transformed to the MRI coordinate system. The initial sensor position refers to the geometrical center of the OPM vapor cell when the sensor is inserted into the helmet with its bottom aligned with the slot bottom. The orientation refers to the radial direction of the slot. The ref.mat (required for Fastrak and the structured-light scanner) and the helmet.ply (required for the laser scanner) are used to complete transform 1.

#### Input data in magnetoencephalography-head coordinate system

The data in the MEG-Head coordinate system is separately collected using each device while the subject was wearing the helmet before/after the OPM-MEG procedure. For Fastrak, the subject’s head points (head points.xlsx), including the left/right pre-auricular points, nasion point, scalp points, and the helmet reference points (helmet ref points.xlsx) are digitized and exported. For the two types of optical scanners, the scanned 3D results (*.ply) are exported.

#### Input data in magnetic resonance imaging coordinate system

The co-registration requires a 3D scalp (MRI.ply), which can be obtained by segmenting the scalp from the acquired MRI data using toolbox such as the SPM (Litvak et al., 2011).

#### Output results

After the two-step transformation, the sensor positions and orientations are transformed from the MEG-Device to the MRI coordinate system (Figure 1B) and the co-registered positions and orientations (sensor_pos_ori.mat) could be exported for solving the forward problem of MEG.

## Tutorial

We illustrate the use of the OMMR toolbox using the tutorial data provided in the toolbox (Supplementary material). The tutorial data for each device were collected from a 26 -year -old, right-handed healthy female. The research protocol was approved by the Ethical Committee of Beihang University, and written informed consent was obtained from the participant. More details about the use of each device and the data collection process can be found in the preceding study (Cao et al., 2021).

### Fastrak

**FIGURE 2:**
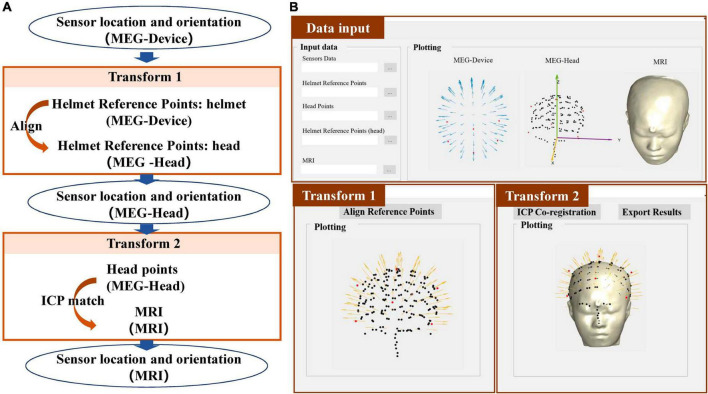
**(A)** Workflow of the co-registration of OPM-MEG and MRI using Fastrak; **(B)** integrated interface for Fastrak.

After the OPM-MEG measurement, the subject still wears the helmet but with the sensors removed. Then, the Fastrak is used to digitize positions of the reference points and the subject’s head points to obtain the data files “helmet ref points.xlsx” and “head points.xlsx,” respectively. The co-registration process using the Fastrak is briefly described in Figure 2A. Transform 1 is completed through the alignment of digitized reference points and the accurate reference points in the designed helmet (ref.mat). Transform 2 is performed by an iterative closest point (ICP) algorithm (Besl and McKay, 1992) which matches the digitized head points and the segmented scalp of the MRI. The Fastrak is also frequently used in the co-registration of SQUID-MEG. In SQUID-MEG, the subjects’ head, fiducial points and HPI coil positions are digitized before the MEG measurement and the HPI coils are then energized during the measurements and localized using the MEG sensors to get the co-registration results. Transform 2 is the same in SQUID-MEG and OPM-MEG while Transform 1 is different. SQUID-MEG localizes HPI coils while OPM-MEG aligns the reference points in transform 1.

The interface of each co-registration step is shown in the Figure 2B. After importing the required data according to Table 1, the data will automatically be plotted, which allows users to check the entered data, and, users can switch between transform 1 and transform 2 sequentially. The alignment and the ICP match will be automatically completed. For transform 1 and 2, the registered sensor positions and orientations will be drawn on the digitized points and segmented scalp of the MRI separately, to enable users to view the matching effect. Finally, the co-registered sensor positions and orientations can be exported in the data file sensor_pos_ori.mat.

### Structured-light scanner

**FIGURE 3:**
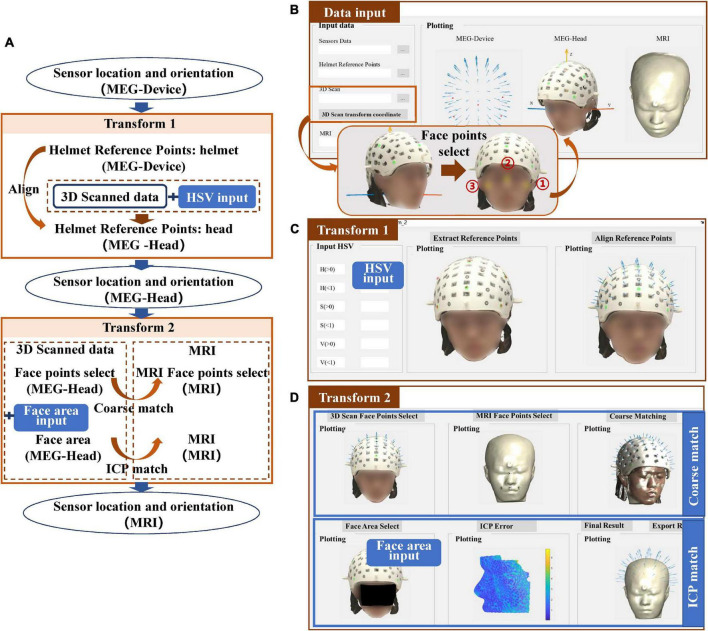
**(A)** Workflow of the co-registration of OPM-MEG and MRI for structured-light scanner; **(B)** interface for importing data; **(C)** interface for transform 1; **(D)** interface for transform 2.

The co-registration process of the structured-light scanner is shown in Figure 3A. The completion of transform 1 depends on the alignment of the helmet reference point, in a similar manner to the transform 1 procedure for Fastrak. However, where it differs, is that the positions of the reference points are obtained using the color extraction method. Transform 2 uses the ICP algorithm to match the face points between the 3D scanned data and the segmented scalp of the MRI.

For the structured-light scanner, the data in the MEG-Head coordinate system is replaced by the scanned 3D images. When importing the 3D images into the toolbox, users need to select three points on the scanned subject’s face to generate the MEG-Head coordinate system in a better view. The requirement of the positions of these points is not strict, however, users need to select the points sequentially following the order in the Figure 3B. The imported data are then displayed visually in the interface.

For transform 1, to extract the helmet reference points in the 3D scanned images, users need to input the HSV parameters to limit the color to the reference points of their own designed helmet, as shown in Figure 3C. For the tutorial data, green markers were used for the helmet thus the HSV parameters were set to H (0.2–0.66), S (0.3–1), V (0.16–1). The “Extract Reference Points” option allows users to check the color extraction results and users also can modify the HSV parameters to achieve the desired visualization. Once the HSV is appropriately set, the alignment of the helmet reference points can be done automatically.

Transform 2 is accomplished by the coarse match and ICP match, where the coarse match simply provides a better initial start for the ICP match. The coarse match requires users to select at least four sets of corresponding points in the MEG-Head and MRI coordinate systems. After clicking the “points select” button, the toolbox will provide an operation hint to guide the users how to select these four sets of points. For the ICP, to improve the efficiency of the co-registration, users need to input the face area manually to limit the candidate match area (Koessler et al., 2011; Bardouille et al., 2012), as shown in Figure 3D. When the face area is selected, the ICP will be performed and the match error will be shown. The co-registration results can then be exported.

### Laser scanner

**FIGURE 4:**
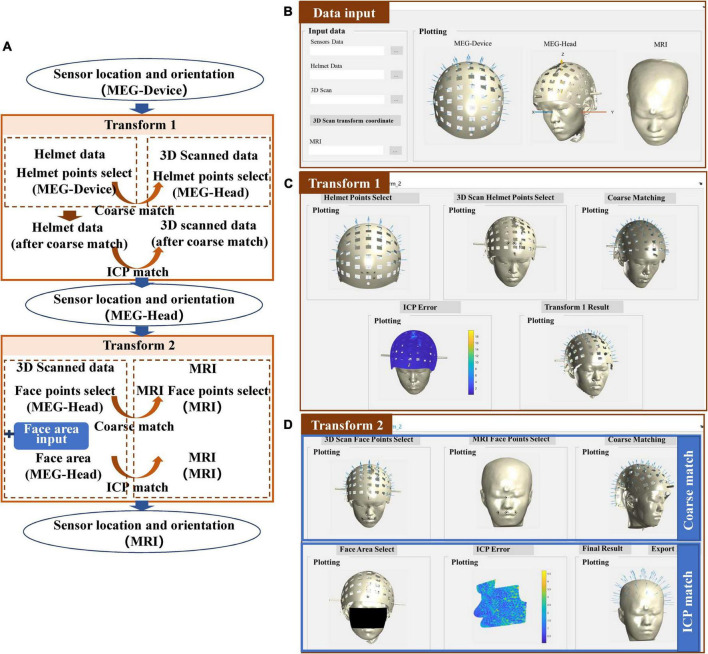
**(A)** Workflow of the co-registration of OPM-MEG and MRI for the laser scanner; **(B)** interface for importing data; **(C)** interface for transform 1; **(D)** interface for transform 2.

The co-registration process of the laser scanner is shown in Figure 4A. Compared to the structured-light scanner, the laser scanner cannot acquire color information of the scanned object thus it cannot use the color threshold to extract the helmet reference points. Instead, it makes use of high 3D reconstruction accuracy, to allow for clear reconstruction of the helmet. Therefore, transform 1 is accomplished by the coarse match and the ICP match between the designed helmet and the scanned helmet. The input data in the MEG-Device coordinate system is replaced by the 3D-designed helmet data, as shown in Figure 4B. The results of transform 1 are quite similar to the match between the scanned face area and the segmented face of the MRI, as shown in Figure 4C. In addition, the match error of the helmet will be shown to allow users to check matching results. Users can then switch to transform 2, which follows the procedure as that of the structured-light scanner, as shown in Figure 4D.

### Depth adjustment

**FIGURE 5:**
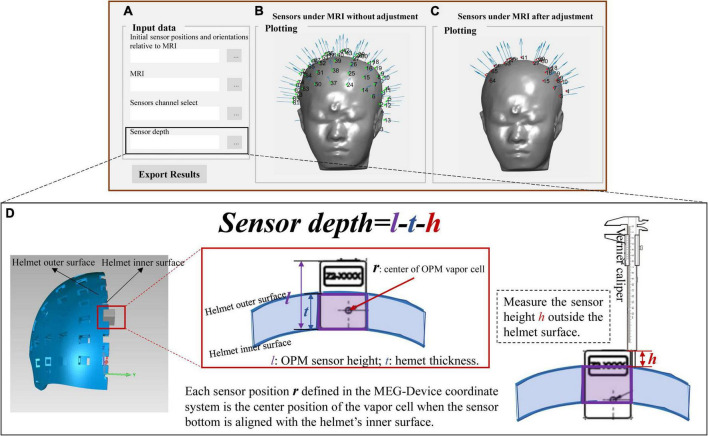
**(A)** Interface for importing data and exporting data; **(B)** the results of co-registration under MRI coordinate system in initial position; **(C)** the final co-registration results; **(D)** the illustration of the sensor depth.

The final output (sensor_pos_ori.mat) after the above co-registration step is the initial sensor positions and orientations relative to the MRI coordinate system. In practical use, users may only use the part of sensor slots and the inserted sensor depths are adjusted to ensure that the sensors are as close as possible to the scalp. In this case, the sensor output should be corrected. We further provide an additional interface (Sensor_select_depth.fig) to allow users to select sensor channels and adjust the sensor depth. Users only need to import the selected sensor number (channel_select.xlsx) and the recorded sensor depth (depth.xlsx); then, the selected and corrected sensor positions and orientations will be outputted, as shown in Figure 5. The sensor depth is defined as the offset between the inserted sensor position and the sensor position defined in the MEG-Device coordinate system, as illustrated in Figure 5D. The sensor position defined in the MEG-Device coordinate system is the center of the OPM vapor cell when the sensor’s bottom is aligned with the slot bottom. The height of the OPM sensor and the thickness of the helmet are denoted as *l* and *t*, respectively, and their values depend on the size of the user’s own OPM sensor and helmet. When inserting the sensor in actual use, users can use a vernier caliper or other measuring tool to record the height *h* of sensor exposed outside the helmet. The sensor depth that should be imported in Figure 5A is depth = *l*-*t*-*h*.

## Discussion

In order to further the applications of OPM-MEG in the research fields, a streamlined GUI-based toolbox called OMMR was developed, to accomplish the co-registration of OPM-MEG and MRI. A tutorial and a set of tutorial data for the toolbox, was provided and the functions and use of the toolbox was illustrated with a step-by-step process description. OMMR is the first toolbox of its kind to provide a full-process of visualized operation of the co-registration of OPM-MEG and MRI, significantly improving the accessibility and ease of use of OPM-MEG. The main advantages of OMMR are that it covers the co-registration methods corresponding to the most commonly used devices and it is simple and convenient to use. The OMMR toolbox is a generalizable toolbox that allows researchers to import their own designed rigid helmet files and follow the tutorial to achieve OPM-MEG and MRI co-registration.

The toolbox is tested using our homemade helmet. Our helmet was designed with 85 sensor slots and three-bolt locking structures to fix the helmet relative to the subject’s head. The helmet was 3D printed using the Lite 600 system with DSM 8000 resin with a printing accuracy of ± 0.2 mm. Our previous work quantified and evaluated the performance of each co-registration method (Cao et al., 2021). In addition, we have applied the most accurate co-registration method, the laser scanner, in real experiments measuring the somatosensory evoked fields (An et al., 2022a,b). The validity of each co-registration method is verified. In practical use, the ground truth of sensor positions and orientations is unknown. To allow users to check the co-registration performance, we further provide a display of the ICP fitting error. In our previous experiments, the average ICP fitting error of the face is less than 2 mm and that of the helmet is less than 0.8 mm. We would suggest that if the ICP error is too large in the user’s experiment, it is better to check the scanned or digitized data quality. Our software directly outputs the co-registered sensor positions and orientations, which can be organized into the sensor configuration for each MEG data format. For example, in FieldTrip, the MEG data is organized in CTF data format, and users just need to replace sensor information in the data.grad file with the co-registered results. In the future, we will try to organize the data into general sensor formats (BIDS) to facilitate usage.

In our toolbox, we provided the co-registration methods for the rigid helmet. It should be noted that although we only provided the sensor measuring the radial components in the tutorial data, it is easy for users to extend it to situations where tri-axis OPMs are used. Users need to determine the tangential directions in their helmet file and then add them to the sensor.mat data file. The co-registration of tangential orientations can also be obtained following the same co-registration process as that of the radial direction. A new co-registration method that uses coils is emerging (Iivanainen et al., 2022). This field is currently growing, and our toolbox will be updated following this growth.

Although we advocate the use of rigid helmets, there are still special requirements for using a flexible cap, especially in clinical research (Duque-Muñoz et al., 2019; Feys et al., 2022). Another advantage of a flexible cap is that it allows the sensors, scalp, and face points to be digitized at the same time (Feys et al., 2022). This will render the transform 1 step obsolete. In co-registration, the less steps, the smaller the errors. Our current toolbox does not support the co-registration with a flexible cap. However, it will be included soon.

## Data availability statement

The original contributions presented in this study are included in the article/Supplementary material. The toolbox, tutorial data, and visualization video is available at https://doi.org/10.5281/zenodo.6958134, further inquiries can be directed to the corresponding author FC, caofuzhi@buaa.edu.cn.

## Ethics statement

The studies involving human participants were reviewed and approved by the Ethical Committee of Beihang University. The patients/participants provided their written informed consent to participate in this study. Written informed consent was obtained from the individual(s) for the publication of any potentially identifiable images or data included in this article.

## Author contributions

FC conceived and designed the study and wrote the first draft of the manuscript. CW, WX, WW, and YY performed the experiments. FC and NA completed the software. XN, MX, and YG supervised the research. XN and NA reviewed and edited the manuscript. All authors read and approved the submitted version.
